# PD-1 and ICOS are coexpressed in T follicular helper cells but define three stages of maturation of T follicular regulatory cells

**DOI:** 10.1126/sciadv.adt8901

**Published:** 2025-07-11

**Authors:** Filipa Ribeiro, Diogo Antunes, Ana R. Pires, José Rino, Beatriz Filipe, Kátia Jesus, Ricardo Correia, Válter R. Fonseca, Saumya Kumar, Luis Graca

**Affiliations:** ^1^ Gulbenkian Institute for Molecular Medicine, Lisboa, Portugal.; ^2^ Faculdade de Medicina, Universidade de Lisboa, Lisboa, Portugal.; ^3^ Centre for Individualised Infection Medicine (CiiM), a joint venture between the Helmholtz Centre for Infection Research (HZI) and Hannover Medical School (MHH), Hannover, Germany.

## Abstract

Humoral responses to infection or vaccination require T cell–B cell interactions. T follicular helper (T_FH_) cells drive germinal center (GC) responses by providing help to B cells, whereas T follicular regulatory (T_FR_) cells regulate them. Both mature GC–located T_FH_ and T_FR_ cells are widely characterized by the expression of ICOS and PD-1. However, although human T_FR_ cells share many phenotypic characteristics with T_FH_ cells, we found that ICOS and PD-1 are up-regulated differently in each. Although T_FH_ cells express these proteins synchronously during maturation, they define three maturation stages in T_FR_ cells. T_FR_ cells in an intermediate maturation stage express ICOS, and it is only at the last stage of differentiation that both molecules are expressed at high levels. Although most T_FR_ cells within the B cell follicle are PD-1^−^, the T_FR_ within the GC are ICOS^+^PD-1^+^. These results show that T_FH_ and T_FR_ cells within human lymphoid tissue follow distinct maturation stages.

## Teaser

T follicular helper and regulatory cells in human lymphoid tissue mature distinctly, defined by PD-1 and ICOS protein levels.

## INTRODUCTION

The findings of T follicular helper (T_FH_) and T follicular regulatory (T_FR_) cells controlling germinal center (GC) reactions in mice prompted the study of T follicular subsets in humans. Whereas T_FH_ cells provide help to B cells (*1*), T_FR_ cells suppress the GC response and limit the overall expansion of antigen-specific B cell clones (*2*–*4*). The absence of T_FR_ cells is related to the emergence of autoimmunity in mice (*5*, *6*). Further studies to better understand the definition and function of human T_FR_ cells are critical, which may help in understanding how GC reactions affect dysregulated immune responses, such as autoimmunity and open avenues for its modulation in the future.

The definition of T_FR_ cells has been controversial, given the diversity of T_FR_ populations with distinct origin (*7*–*11*), and the fact that the T_FR_ cell biology has not been described with the same detail as T_FH_ cells. Because of the absence of lineage-defining markers exclusive to T_FR_ cells, the phenotype and differentiation of T_FR_ cells have been frequently studied by extrapolating T_FH_ cell data. CXCR5 has been a key defining follicular marker for T_FH_ and T_FR_ cells as its expression correlates with the follicular location in both cases (*12*, *13*). Moreover, anatomical positioning of T_FH_ cells within GC is also governed by the signaling of ICOS (*14*, *15*), which supports CXCR5^+^ T cell migration toward the follicle and PD-1 by promoting the retention of T_FH_ cells in the GC (*16*). Therefore, these two receptors have been widely used, together with CXCR5, to define GC T_FH_ cells in both humans and mice. It has been assumed that T_FR_ cells follow a differentiation process similar to that of T_FH_ cells, and therefore, the same markers have been used to identify both cell subsets (*17*). Besides the molecules associated with regulatory T (T_reg_) cells (such as FOXP3 and/or CD25), a combination of two proteins is commonly used to identify lymphoid tissue T_FR_ cells: CXCR5 with either ICOS or PD-1, which are often used interchangeably.

Nevertheless, the follicle positioning of T_FR_ cells in human lymph nodes was shown to be determined by CXCR5 expression but not by PD-1 expression as for T_FH_ cells (*13*). Thus, we hypothesized that PD-1 and ICOS expression does not follow the same profile in T_FH_ and T_FR_ cells, and consequently, they cannot be used interchangeably to define T_FR_ cells as they are for T_FH_ cells. This was successfully demonstrated in the current study.

A major constraint in the field of T_FR_ cells is the difficulty in isolating human T_FR_ cells from lymphoid tissues with a high degree of purity due to the lack of surface markers that discriminate this cell population from others. One of the major challenges of studying human T_FR_ cells is the absence of known cell surface markers that can uniquely define them, without being also present in T_FH_, CD25^+^ interleukin-10 (IL-10)–producing T follicular cells (IL-10 TF), or T_reg_ cells. A better understanding of ICOS and PD-1 expression dynamics in human lymphoid tissue T_FR_ cells allowed us to explore innovative strategies to isolate this cell population for potential downstream analysis.

## RESULTS

### ICOS and PD-1 are expressed differently in T_FH_ and T_FR_ cells within human secondary lymphoid tissue

We analyzed 50 tonsil samples by flow cytometry and applied a permissive strategy, based only on the expression of FOXP3 and CXCR5, to define T_FH_ (CD4^+^FOXP3^−^CXCR5^+^CD25^−^) and T_FR_ cells (CD4^+^FOXP3^+^CXCR5^+^). Using this strategy, the IL-10 TF cells (CD4^+^FOXP3^−^CXCR5^+^CD25^+^) were not included in the study (*18*, *19*).

**Fig. 1.**
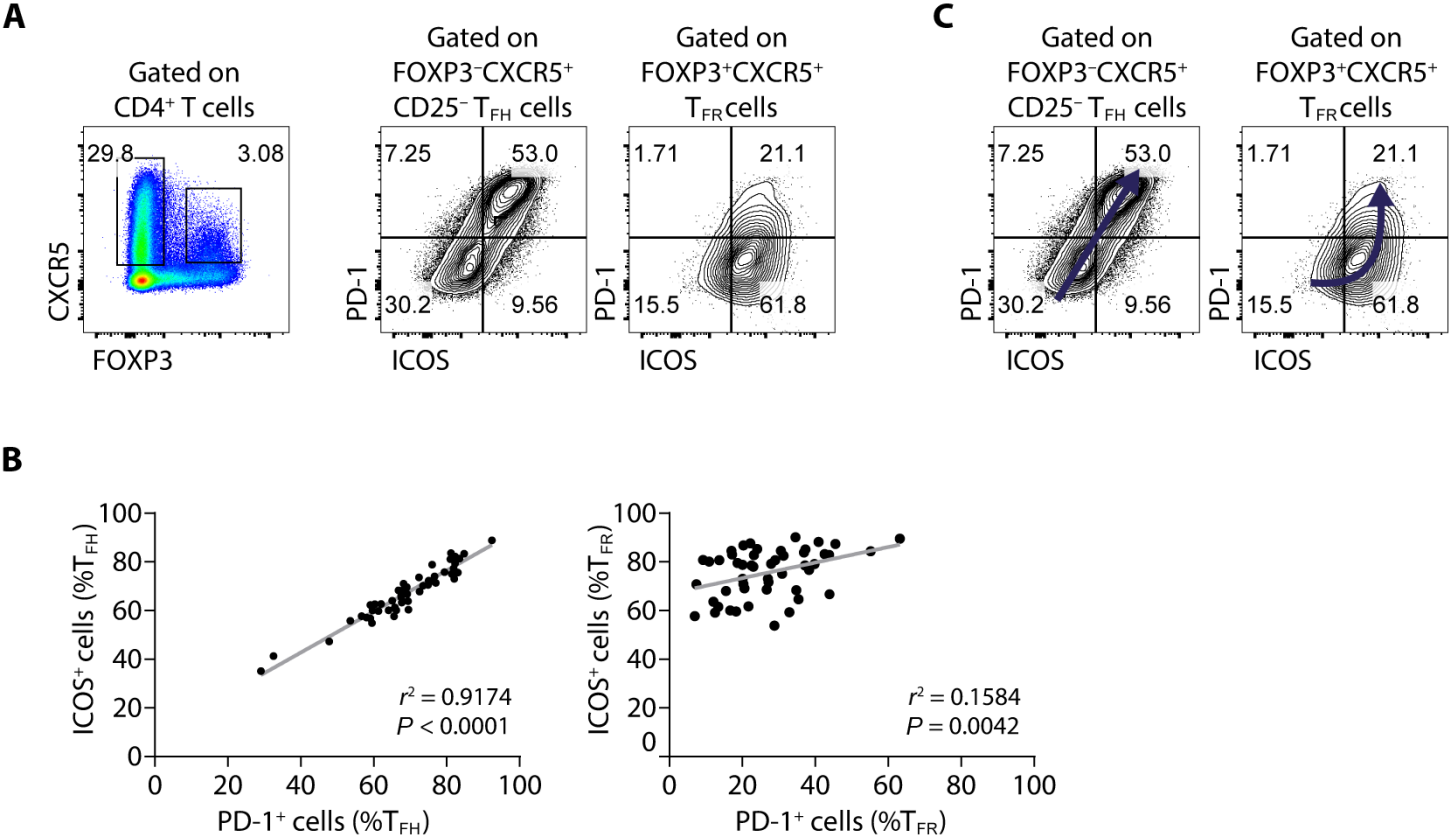
ICOS and PD-1 are expressed differently in tissue T_FH_ and T_FR_ cells in humans. (**A**) Presence of ICOS and PD-1 on the surface of T_FH_ (CD4^+^FOXP3^−^CXCR5^+^CD25^−^) and T_FR_ cells (CD4^+^FOXP3^+^CXCR5^+^) in human tonsils. Density plot showing the gate used to define T_FH_ cells (CXCR5^+^FOXP3^−^) and T_FR_ cells (CXCR5^+^FOXP3^+^) and two representative contour plots showing the distribution of PD-1 and ICOS among the T_FH_ and T_FR_ gates. (**B**) Relationship of the percentage of ICOS^+^ and PD-1^+^ cells within T_FH_ (left) and T_FR_ cells (right) in tonsil (*n* = 50; linear regression). (**C**) Diagram with a model for the trajectory of T_FH_ and T_FR_ maturation defined by PD-1 and ICOS, where T_FH_ cells transit from a double-negative to a double-positive stage (left), whereas T_FR_ cells have three stages: double-negative, ICOS^+^PD-1^−^, and lastly double-positive.

Within the cell subsets defined as T_FH_ and T_FR_, we explored the presence of the activation markers ICOS and PD-1 known to be characteristic of the most mature cells within the GC (*17*) (Fig. 1A). Tonsil T_FH_ and T_FR_ cells have a clearly different PD-1/ICOS profile: Whereas the expression of ICOS and PD-1 correlates almost perfectly in T_FH_ cells, appearing to be interchangeable, this is not the case for T_FR_ cells (Fig. 1B). In the latter, the correlation between the ICOS and PD-1 is weak, indicating that these proteins cannot be used interchangeably to identify T_FR_ cells as they are for T_FH_ cells. We therefore hypothesize that, although T_FH_ cells mature from ICOS^−^PD-1^−^ toward an ICOS^+^PD-1^+^ phenotype, acquiring the two receptors simultaneously and at a similar rate, the maturation of T_FR_ cells from an ICOS^−^PD-1^−^ to the most mature ICOS^+^PD-1^+^ stage occurs in two stages. The first stage involves the up-regulation of ICOS, followed by a subsequent increase in the production of PD-1 (Fig. 1C).

### Human T_FH_ and T_FR_ cells follow different maturation axes

To test our hypothesis, we analyzed the expression of the transcription factor BCL6, known to be up-regulated by the mature GC cells (*20*), and CD45RO, also known to be more expressed in mature T cells (*21*). It is noteworthy that CD45RO is a marker of activated T cells and, given that T follicular cells are already in an activated state, it is reasonable to anticipate some production of this protein even in less mature follicular cells.

**Fig. 2.**
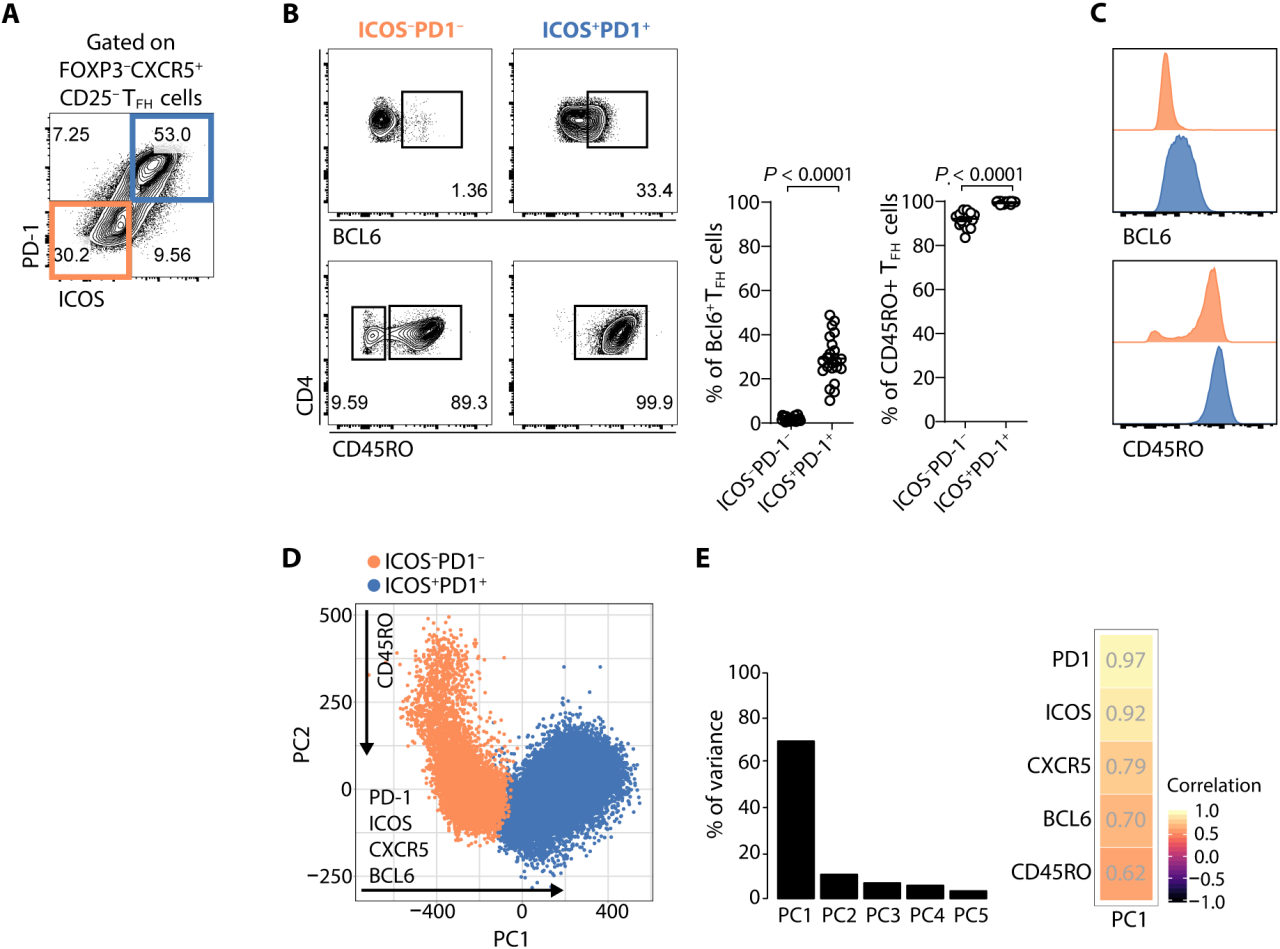
ICOS and PD-1 expression increase in a coordinated way throughout the maturation of human T_FH_ cells. (**A**) Identification of the ICOS^−^PD-1^−^ (orange) and ICOS^+^PD-1^+^ (blue) subsets within tonsil CD4^+^FOXP3^−^CXCR5^+^CD25^−^ T_FH_ cells by flow cytometry. (**B**) Frequency of cells positive for BCL6 and CD45RO within the ICOS^−^PD-1^−^ and ICOS^+^PD-1^+^ T_FH_ subsets. Representative plots (left) and pooled data (right) (*n* = 22). Error bars represent means ± SEM. Paired *t* test and Wilcoxon matched-pairs signed-rank test were applied to the BCL6 and CD45RO graphs, respectively. (**C**) Histograms of the distribution of BCL6 and CD45RO among ICOS^−^PD-1^−^ T_FH_ cells (orange) and ICOS^+^PD-1^+^ T_FH_ cells (blue). (**D**) Visualization of the PCA from the fluorescence values of each flow cytometry marker from one tonsil. Cell subsets in the PCA are colored as indicated. (**E**) Percentage of variance of the data explained by the first five principal components (left) and heatmap of the correlation values for each of the markers explained by PC1 (right).

In T_FH_ cells, two major populations were considered: ICOS^−^PD-1^−^ and ICOS^+^PD-1^+^ cells (Fig. 2A and fig. S1). The ICOS^+^PD-1^+^ population represents the most mature T_FH_ cells, characterized by a predominant coexpression of BCL6 and CD45RO, that are present at high concentrations (as inferred from the fluorescence intensity) (Fig. 2, B and C). By extracting the fluorescence intensity of seven markers used in the flow cytometry analysis and using principal components analysis (PCA), we were able to explore the relationship between all markers at the same time. The PCA of T_FH_ cells shows that PC1 is the component that contributes the most to the variance (69.70%) and is therefore sufficient to explain the linear maturation of T_FH_ cells (Fig. 2, D and E). PD-1 and ICOS are the proteins that contribute the most to PC1 and with a similar influence (0.97 and 0.92, respectively) (Fig. 2E). PC2 is not shown as it mainly segregates CD45RO^+^ and CD45RO^−^ T_FH_ populations (fig. S2).

**Fig. 3.**
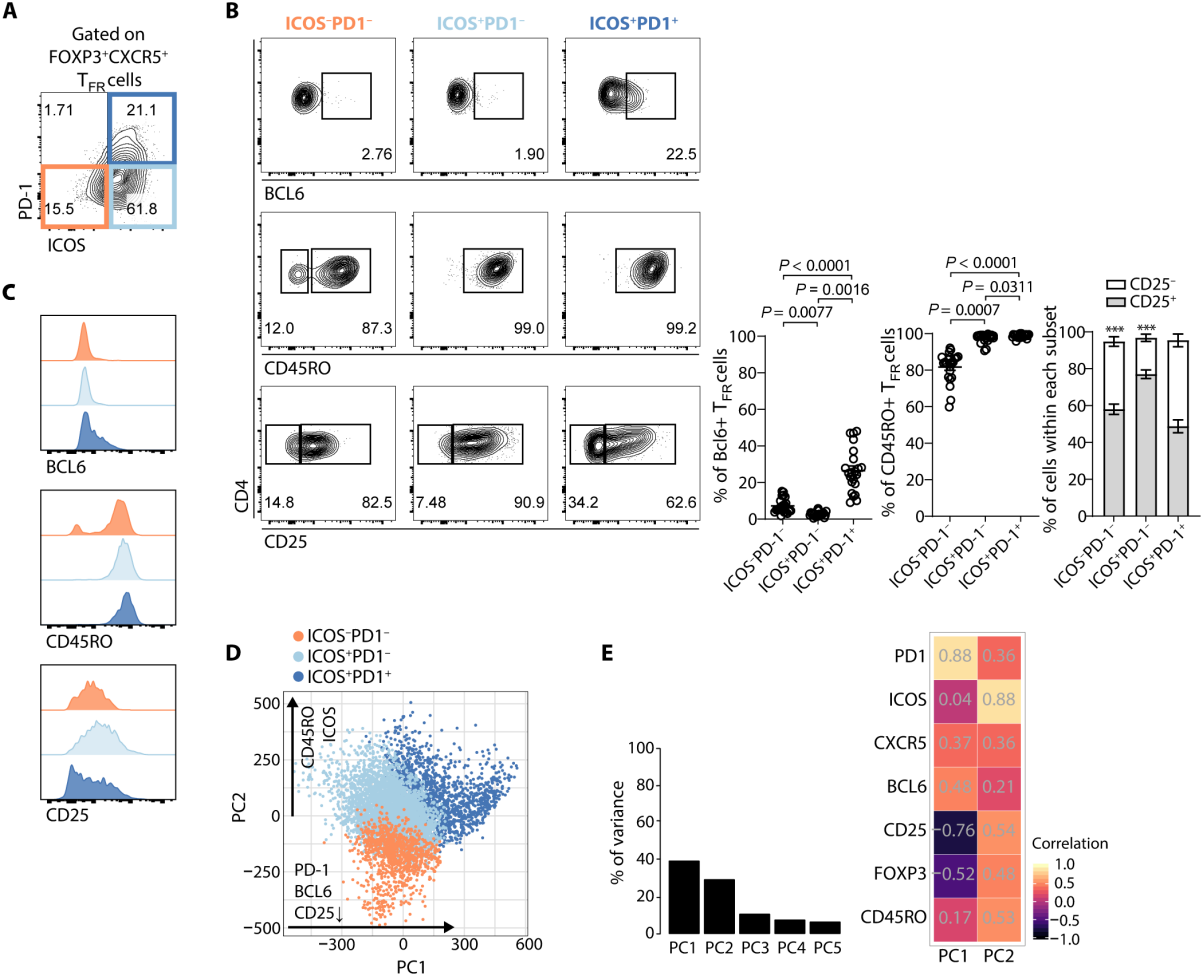
The ICOS^+^PD-1^−^ population represents T_FR_ cells in an intermediate maturation stage. (**A**) Representative contour plot showing the identification of ICOS^−^PD-1^−^ (orange), ICOS^+^PD-1^−^ (light blue), and ICOS^+^PD-1^+^ (blue) subsets of tonsil CD4^+^FOXP3^+^CXCR5^+^ T_FR_ cells by flow cytometry. (**B**) Frequency of cells expressing BCL6, CD45RO, and CD25 within the ICOS^−^PD-1^−^, ICOS^+^PD-1^−^, and ICOS^+^PD-1^+^ T_FR_ subsets. Representative plots (left) and pooled data (right) are shown (*n* = 22). Error bars represent means ± SEM. Friedman test with Dunn’s multiple comparisons test was applied to the BCL6 and CD45RO graphs. For CD25 analysis, ***P < 0.001 using two-way ANOVA with Bonferroni’s multiple comparison test. (**C**) Histograms of BCL6, CD45RO, and CD25 distribution among ICOS^−^PD-1^−^ T_FR_ cells (orange), ICOS^+^PD-1^−^ T_FR_ cells (light blue), and ICOS^+^PD-1^+^ T_FH_ cells (blue). (**D**) Visualization of the PCA from fluorescence values of each flow cytometry marker. The position of the T_FR_ cells from the three subsets within the PCA is indicated by color. (**E**) Percentage of variance of the data explained by the first five principal components (left) and heatmap of the correlation values for each marker explained by PC1 and PC2 (right).

Next, we analyzed the T_FR_ population using the same approach. T_FR_ cells can be subdivided into three populations according to the PD-1/ICOS axis: ICOS^−^PD-1^−^, ICOS^+^PD-1^−^, and ICOS^+^PD-1^+^ cells (Fig. 3A and fig. S1). Similar to T_FH_ cells, the ICOS^+^PD-1^+^ T_FR_ population contains the most mature cells as BCL6 and CD45RO are more abundant compared to the other subpopulations (Fig. 3, B and C). Within this population, BCL6^+^ cells exhibit the highest CXCR5 expression, further reinforcing their advanced maturation within the GC (fig. S3). In addition, the expression of CD25 (IL-2 receptor α chain) was also assessed in the different compartments. It is established that T_FR_ cells lose CD25 in the last stages of their differentiation as they become mature GC T_FR_ cells (*19*, *22*–*24*). The ICOS^+^PD-1^+^ T_FR_ population comprises a greater frequency of CD25^−^ cells, also supporting that this T_FR_ compartment contains the most mature T_FR_ cells (Fig. 3, B and C). According to the production of BCL6, CD45RO, and CD25, T_FR_ cells seem to display ICOS on their surface before up-regulating PD-1 and reaching a fully mature state. However, we observed a substantial number of CD25^−^ cells within the ICOS^−^PD-1^−^ T_FR_ compartment that was not expected, given their overall less mature phenotype (namely, regarding PD-1 and ICOS expression) (Fig. 3, B and C, and fig. S4). This could be explained by the possible existence of some CD25^−^ T_reg_ cells even outside the GC (*25*, *26*).

Unlike what we observed for T_FH_ cells, the variance of the T_FR_ cell data is not explained by only one principal component, but with both PC1 and PC2 having a similar impact on the variance (38.97 and 28.84%, respectively) (Fig. 3, D and E). The correlation matrix of the expression of the different markers in T_FR_ cells shows that, whereas PC-1 explains the up-regulation of PD-1 and BCL6 and the down-regulation of CD25, PC2 describes the up-regulation of ICOS and CD45RO (Fig. 3E). The up-regulation of CXCR5 is described by both PC1 and PC2 (Fig. 3E). Therefore, whereas T_FH_ maturation is linear and explained by only one axis, the T_FR_ cell maturation is explained by two different axes, with one of the axes associated with PD-1 and the other with ICOS. Consequently, ICOS and PD-1 cannot be used interchangeably to identify T_FR_ cells as three populations are defined based on the expression of these receptors.

### ICOS^+^PD-1^+^ T_FR_ cells are located specifically within the GC

**Fig. 4.**
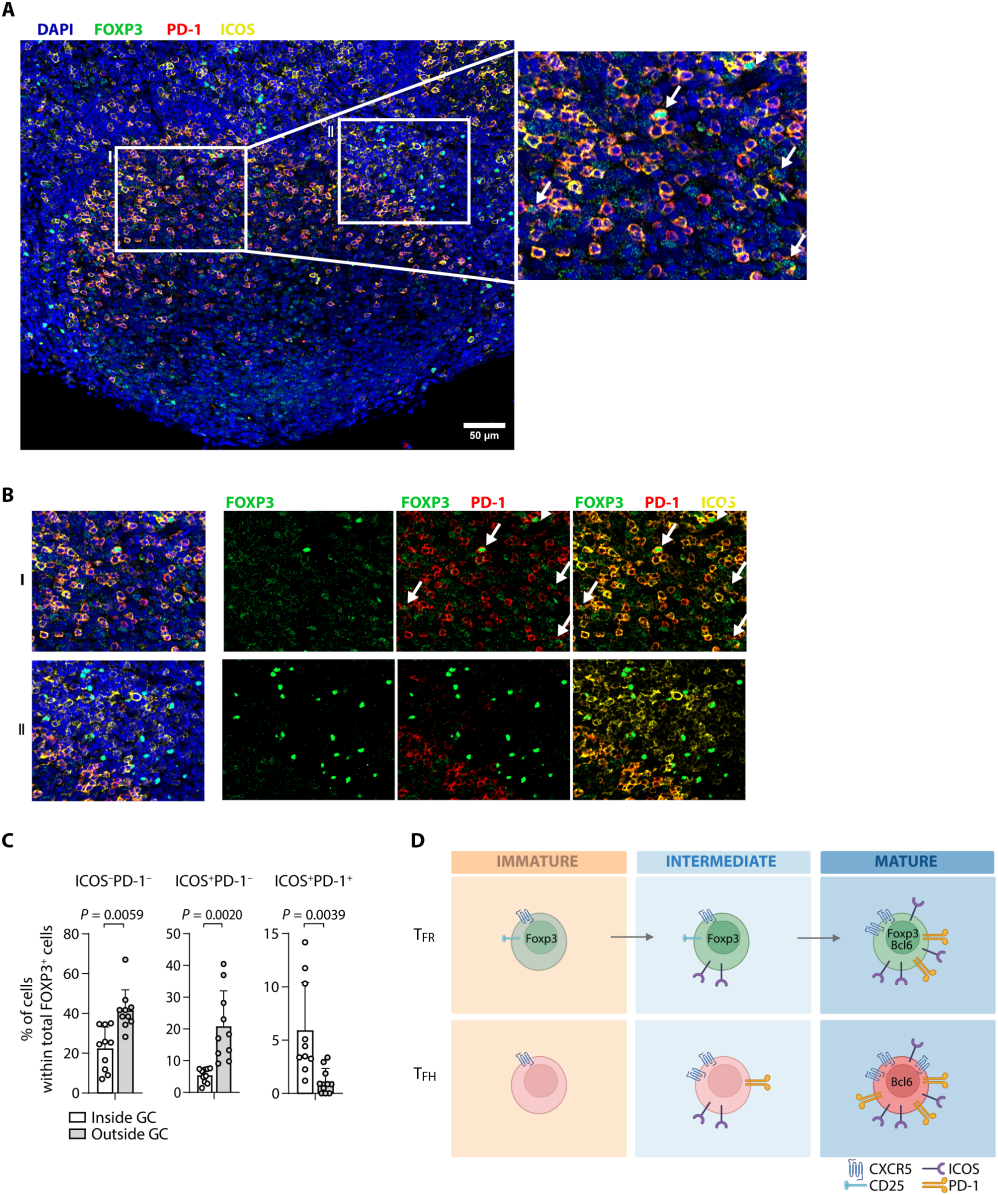
ICOS^+^PD-1^+^ T_FR_ cells localize specifically within the GC. (**A**) Representative image of the immunofluorescence microscopy of human tonsils stained for DAPI (blue), FOXP3 (green), ICOS (yellow), and PD-1 (red). Individual image panels were stitched together to reconstruct the full sample area using tile imaging and *z*-stack acquisition. Outlined area I is represented as an enlarged image on the right, in which ICOS^+^PD-1^+^FOXP3^+^ cells are identified (white arrows). (**B**) Outlined areas I and II indicated in (A). Data are representative of tonsil sections from four healthy children (see fig. S6 for another example). (**C**) Quantification of ICOS^−^PD-1^−^, ICOS^+^PD-1^−^, and ICOS^+^PD-1^+^ T_FR_ cells within (white bars) and outside (gray bars) the GC. Data are pooled from two to three images per tonsil of four healthy children (*n* = 10). Error bars represent means ± SEM. Wilcoxon matched-pairs signed-rank test was applied. (**D**) Model of T_FH_ and T_FR_ cell maturation in human tissue, given by the expression of ICOS, PD-1, CXCR5, BCL6, and CD25. Created in BioRender. I. Biorender (2025), https://BioRender.com/1cjps16.

To consolidate our previous findings, confocal images were acquired from human tonsils. As expected, PD-1 was highly expressed in the GC (Fig. 4A). In line with the hypothesis that PD-1 expression is restricted to the most mature T_FR_ cells, we found few PD-1^+^ cells among the FOXP3^+^ T_FR_ cells, but those PD-1^+^ T_FR_ cells also expressed ICOS and were located within the GC (Fig. 4, B and C, and fig. S5). Conversely, FOXP3^+^ cells found outside the GC, namely, in the follicle, were either ICOS^+^ or did not express either ICOS or PD-1 (Fig. 4, B and C, and fig. S5). These findings align with our earlier observations using flow cytometry indicating that ICOS^+^PD-1^+^ T_FR_ cells are a minor population, which likely represent the most mature subset, and are therefore primarily located within the GC (Fig. 4D). Whereas a substantial proportion of FOXP3^+^ cells in the GC are ICOS^−^PD-1^−^, the ICOS^+^PD-1^+^ subset is enriched in the GC, supporting the notion that their maturation is associated with this specific location.

### The tonsil CD4^+^CD25^+^CXCR5^int^ICOS^int^ population contains a high frequency of FOXP3^+^ cells, representing an innovative approach to isolating T_FR_ cells from human lymphoid tissue

Whereas human circulating T_FR_ cells can be readily isolated based on the expression of CXCR5 and CD25 (*27*), the isolation of T_FR_ cells from human lymphoid tissue has been challenging and an obstacle in studying their suppressive function. No unique extracellular markers are known to define lymphoid tissue T_FR_ cells unequivocally allowing the isolation of a pure T_FR_ cell population (containing a high frequency of FOXP3^+^ cells). Our data on the expression of ICOS and PD-1 described above prompted us to explore alternative strategies to isolate T_FR_ cells from human tissue.

**Fig. 5.**
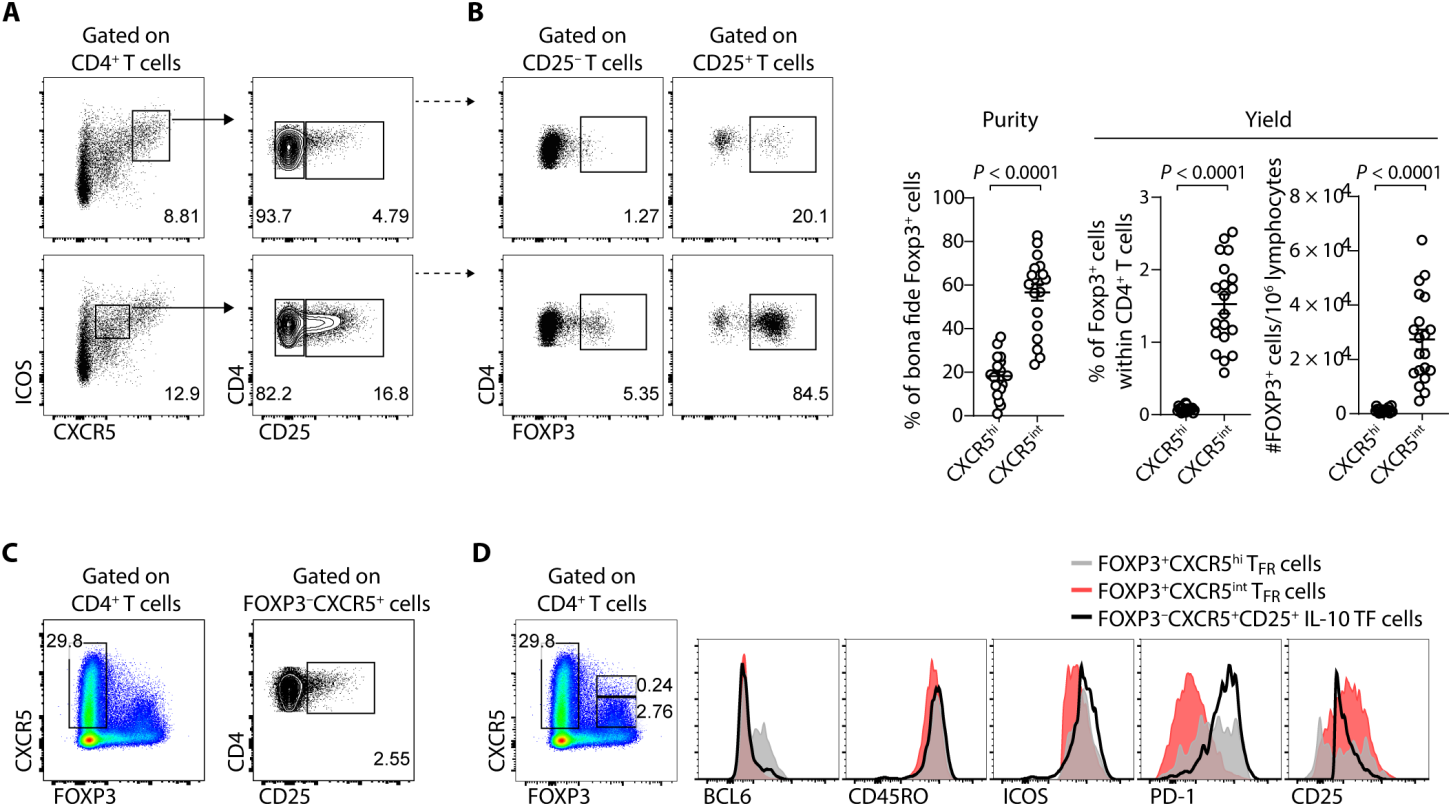
The CD4^+^CD25^+^CXCR5^int^ICOS^int^ cell population from human tonsils contains a high frequency of FOXP3^+^ cells. (**A**) Comparison of two alternative gating strategies used for sorting T_FR_ cells from lymphoid tissue, selecting either CXCR5^hi^ICOS^hi^ cells (top; conventional strategy) or CXCR5^int^ICOS^int^ cells (bottom; proposed strategy). (**B**) Analysis of the frequency of FOXP3^+^ T_FR_ cells within the hypothetical isolated population using the strategies described in (A): CXCR5^hi^ICOS^hi^CD25^−^ (top left), CXCR5^hi^ICOS^hi^CD25^+^ (top right), CXCR5^int^ICOS^int^CD25^−^ (bottom left), and CXCR5^int^ICOS^int^CD25^+^ (bottom right). Representative plots (left) and pooled data (right) (*n* = 20). Error bars represent means ± SEM. Paired *t* test was applied. (**C**) Representative plots showing CD4^+^FOXP3^−^CXCR5^+^CD25^+^ IL-10 TF cells. (**D**) Expression of BCL6, CD45RO, ICOS, PD-1, and CD25 by CXCR5^hi^ T_FR_ cells (gray), CXCR5^int^ T_FR_ cells (red), and IL-10 TF cells from human tonsils (black line).

The most common way to isolate human T_FR_ cells from lymphoid tissue relies on the selection of CD4^+^CD25^+^CXCR5^hi^ICOS^hi^ cells. Nevertheless, human tonsils contain a very low percentage of CD25^+^ cells among the CD4^+^CXCR5^hi^ICOS^hi^ cells (Fig. 5A) and, within those, only ~20% of the cells are positive for FOXP3 (Fig. 5B), leading to very few “true” T_FR_ cells (i.e., FOXP3^+^ cells) isolated using this gating strategy. In addition, this strategy does not exclude the CD25^+^FOXP3^−^ IL-10 TF cells, which also have suppressive function, and are numerically similar to the T_FR_ cells in this population (*11*, *18*). Furthermore, despite the abundance of ICOS, most T_FR_ cells do not express high levels of CXCR5; instead, they express intermediate levels (fig. S7). Consequently, although selecting CXCR5^hi^ cells allows capturing the most mature T_FR_ cells (albeit among many “contaminant” Foxp3^−^ cells), this strategy would not yield a representative proportion of the global T_FR_ cell population. Alternatively, based on our previous observations, CD4^+^CXCR5^int^ICOS^int^ cells contain a considerably higher percentage of CD25^+^ cells (Fig. 5A), which, in turn, is reflected in a markedly higher proportion of selected FOXP3^+^ cells (Fig. 5B). Thus, we propose to select CD4^+^CD25^+^CXCR5^int^ICOS^int^ cells to isolate from human lymphoid tissue a population with a greater purity of T_FR_ cells (assessed by the frequency of the FOXP3-expressing cells in the sorted population) and a better yield of T_FR_ cells (Fig. 5B).

The proposed strategy offers a significant improvement in isolating T_FR_ cells with intermediate levels of CXCR5 expression. This approach may result in the exclusion of cells with higher CXCR5 expression, which also exhibit elevated levels of ICOS, PD-1, and BCL6. This excluded subset represents a minor yet critical population, encompassing the most mature CD25^−^ T_FR_ cells. The generally used gating strategy, by selecting CD4^+^CD25^+^CXCR5^hi^ICOS^hi^ cells, contains a substantial proportion of the recently described IL-10–producing T follicular (IL-10 TF) cells (*11*, *18*). Although IL-10 TF cells lack FOXP3 expression, they differ from prototypic T_FH_ cells by expressing CD25 (Fig. 5C). However, IL-10 TF cells closely resemble FOXP3^+^CXCR5^hi^ T_FR_ cells in terms of the presence of ICOS and PD-1 (Fig. 5D), as well as their regulatory function of suppressing T cell proliferation mediated by IL-10 (*18*). Therefore, given the increased frequency of FOXP3^−^ cells (Fig. 5B), targeting the CD4^+^CD25^+^CXCR5^hi^ICOS^hi^ cell population appears to be a promising strategy for isolating IL-10 TF cells.

The most mature FOXP3^+^CXCR5^hi^ T_FR_ cells show higher expression of BCL6 and less expression of CD25 compared to FOXP3^+^CXCR5^int^ T_FR_ cells (Fig. 5D). These observations are consistent with previous studies suggesting that lymphoid tissue T_FR_ cells down-regulate CD25 and increase CXCR5 expression as they mature, both in mice and humans (*23*, *24*). However, CD25 needs to be used in a T_FR_ cell isolation strategy based on CXCR5 and ICOS markers to exclude the T_FH_ cells (CD25^−^) that are present in vast excess within the CXCR5^hi^ICOS^hi^ gate. Consequently, the most mature CD25^−^ T_FR_ cells are also absent from a sorting strategy based on CXCR5^hi^ICOS^hi^ cells. In conclusion, although an ideal approach for isolating mature CD25^−^ T_FR_ cells remains to be defined, the proposed strategy based on CD4^+^CD25^+^CXCR5^int^ICOS^int^ greatly increases the purity and number of T_FR_ cells isolated from human lymphoid tissue, although with a bias toward capturing the intermediate maturation stages.

### Lymphoid tissue T_FR_ cells have greater suppressive function than circulating T_FR_ cells

**Fig. 6.**
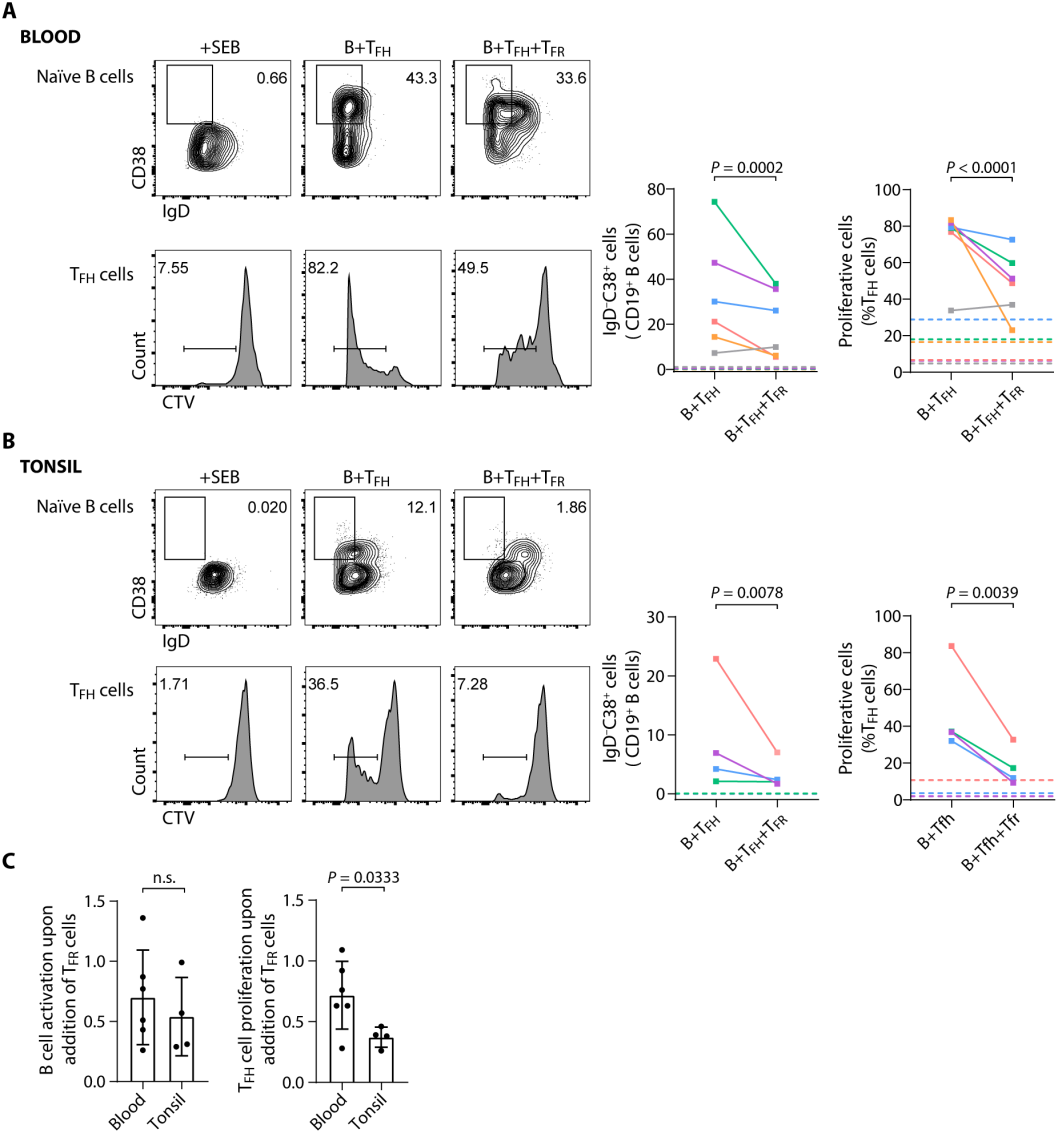
Suppressive function of circulating and tonsil T_FR_ cells. To assess the suppressive capacity of T_FR_ populations, cocultures of T_FR_ cells (from peripheral blood or human tonsils) with naïve B cells and T_FH_ cells from the same donor were assessed after 5 days of stimulation with SEB. (**A**) In cultures with T_FR_ cells isolated from the peripheral blood (sorted as CD4^+^CD25^+^CXCR5^+^), up-regulation of CD38 and down-regulation of IgD by naïve B cells (top) and proliferation of T_FH_ cells measured by CTV dilution (bottom) were analyzed. Representative plots (left) and pooled data (right) are shown (*n* = 6). (**B**) In cocultures of tonsil T_FR_ cells (sorted as CD4^+^CD25^+^ICOS^int^CXCR5^int^), up-regulation of CD38 and down-regulation of IgD by naïve B cells (top) and proliferation of T_FH_ cells measured by CTV dilution (bottom) were analyzed. Representative plots (left) and pooled data (right) are shown (*n* = 4). In (A) and (B), each donor is represented by a unique color and each dot represents the mean of technical replicates within the same donor. Horizontal dashed lines represent controls (B cells + SEB; or T_FH_ + SEB). Statistical significance was assessed using the Wilcoxon matched-pairs signed-rank test. (**C**) Comparison of the ability of T_FR_ cells isolated from the peripheral blood and human tonsil to suppress B cell activation and T_FH_ cell proliferation, applying the ratio between cocultures with and without T_FR_ cells described above [(B+T_FH_+T_FR_):(B+T_FH_)] (*n* = 6 for blood and *n* = 4 for tonsil; unpaired Mann-Whitney *U* test); n.s., not significant.

To address the suppressive function of lymphoid tissue T_FR_ cells, we performed in vitro cocultures to measure their ability to suppress B cell activation and T_FH_ cell proliferation. We performed the same assays with circulating T_FR_ cells. We found that, in line with what was previously reported both in mice and humans (*27*, *28*), circulating T_FR_ cells (isolated as CD4^+^CD25^+^CXCR5^+^) can suppress B cell activation, as measured by CD38 up-regulation, and T_FH_ cell proliferation (Fig. 6A). Tonsil T_FR_ cells were sorted from the tissue using the strategy described above, as CD4^+^CD25^+^CXCR5^int^ ICOS^int^. Therefore, we anticipate a bias toward an intermediate maturation stage (and not including the most mature CD25^−^ T_FR_ cells), but in the absence of the putative contaminating CD25^+^ IL-10 TF cells. We found that the lymphoid tissue T_FR_ cells can suppress the activation of B cells and proliferation of T_FH_ cells (Fig. 6B). Furthermore, the tonsil T_FR_ cells, even devoid of the most mature population, could suppress the proliferation of T_FH_ cells more efficiently than circulating T_FR_ cells (Fig. 6C).

## DISCUSSION

There is limited knowledge regarding the differentiation and function of human T_FR_ cells within lymphoid tissue. Our results show that ICOS and PD-1 are useful surface markers to distinguish different developmental states of T_FR_ cells. These regulatory cells up-regulate ICOS and PD-1 in a sequential way as they mature: In an intermediate maturation stage, T_FR_ cells display ICOS in the absence of PD-1, whereas only the most mature T_FR_ cells coexpress both ICOS and PD-1.

We examined a panel of 50 human tonsils with a comprehensive flow cytometry approach to characterize the expression of ICOS and PD-1 on the maturation of T_FH_ and T_FR_ cells. We identified three main subsets of T_FR_ cells—PD-1^−^ICOS^−^, PD-1^−^ICOS^+^, and PD-1^+^ICOS^+^—with different characteristics regarding the expression of canonical follicular and GC markers. This observation is markedly distinct from what is observed in T_FH_ cells, where we found a strong linear correlation between cells expressing PD-1 and those expressing ICOS. The correlation is so precise that the two molecules can be used interchangeably to define the maturation trajectory of T_FH_ cells. The PCA further supports a linear maturation of T_FH_ cells: At their most immature stage, T_FH_ cells are PD-1^−^ICOS^−^, and they acquire PD-1, ICOS, and BCL6 in a coordinated throughout the maturation process. Our previous work using a pseudo-time algorithm to infer the developmental trajectory of human T_FH_ cells based on single-cell transcriptomics datasets showed that the increase in gene expression of ICOS and PDCD1 (PD-1) occurs almost simultaneously (*19*).

The maturation of human T_FR_ cells seems to have another layer of complexity. The distribution of the analyzed proteins along the T_FR_ maturation trajectory is not linear, as suggested by our PCA. The lack of expression of the GC marker BCL6 suggests that PD-1^−^ICOS^−^ T_FR_ cells have an immature phenotype similar to that described for their counterparts in blood (*27*). Conversely, the PD-1^+^ICOS^+^ subset of T_FR_ cells can be considered as the most mature, given the abundance of BCL6 and CD45RO, but not CD25. CD25 is lost in the most mature GC T_FR_ cells (*19*, *23*, *24*). Nevertheless, numerically, very few cells correspond to the most mature CD25^−^ T_FR_ population displaying high levels of both ICOS and PD-1. This finding aligns with the research conducted by Sayin and colleagues, who demonstrated that, in contrast to T_FH_ cells, most T_FR_ cells in human mesenteric lymph nodes do not express PD-1 (*13*). Furthermore, they observed that PD-1^+^ T_FR_ cells exhibited higher levels of ICOS (*13*). Our previous observations in tonsil, using single-cell RNA sequencing, also support this pattern as the small proportion of T_FR_ cells found to express PDCD1 (PD-1) likely represented the most mature T_FR_ cells (*19*).

T_FR_ cells in an intermediate maturation stage express ICOS but little PD-1. A recent study in mice has demonstrated that ICOS is required for generation of T_FR_ cells by indirectly promoting the expression of BCL6 and CXCR5, proving to be essential in the early stages of T_FR_ cell development (*29*). In addition, ICOS may also be important in the later stages of T_FR_ cell function, as supported by research demonstrating that blocking its signaling results in the expansion of autoreactive B cells and increased autoantibody titers (*29*). Conversely, PD-1 inhibits the activation and proliferation of T_FR_ cells in mouse lymph nodes and blood, thereby suppressing the immune response (*30*). In addition, PD-1 expression in the GC seems of utmost importance because it regulates selection and survival, affecting the quantity and quality of long-lived plasma cells (*31*). Although these studies were conducted in mice, they suggest that T_FR_ cells can regulate the immune response by limiting excessive B cell responses and preventing autoimmunity through PD-1. These observations highlight the potential functional significance of the acquisition of PD-1 by fully mature T_FR_ cells.

The distinct location of T_FR_ cells at different maturation stages within the follicle holds key importance. A previous study revealed that PD-1^+^ T_FH_ cells were particularly enriched in GCs, but the relationship between PD-1 surface expression and T_FR_ cell location was less evident (*13*). In addition, it was reported that PD-1^+^ T_FR_ cells display superior IL-10 production compared to PD-1^−^ T_FR_ cells and T_reg_ cells (*13*). Here, we show that ICOS^+^PD-1^+^ T_FR_ cells, as the most mature population, preferentially locate within the GC whereas T_FR_ cells lacking PD-1 expression are mostly found outside the GC.

Part of the limited understanding of T_FR_ cell function in humans can be attributed to the challenge of isolating these cells for in vitro functional studies as there are no known surface proteins allowing their precise sorting. Our findings on PD-1 and ICOS expression led us to explore an alternative strategy for flow cytometry isolation of T_FR_ cells. By selecting CD4^+^CD25^+^CXCR5^int^ICOS^int^ T_FR_ cells, we were able to obtain a significantly enriched population in FOXP3^+^ T_FR_ cells and devoid of CD25^+^ IL-10 TF cells to assess the suppressive function of tissue T_FR_ cells in vitro. We found that tonsil T_FR_ cells have greater suppressive function than circulating T_FR_ cells. These observations are consistent with a previous study showing that circulating T_FR_ cells, being immature, are not yet fully competent in their suppressive ability (*27*). Recently, CD38 was shown to discriminate T_FH_-derived T_FR_ cells from T_reg_-derived T_FR_ cells (*10*). Although the CD38^+^ T_FH_-derived T_FR_ cells represent only about 30% of the overall T_FR_ population, they comprise about 70% of the T_FR_ cells in the GC and only 7% of the T_FR_ cells in the B cell follicle (*10*) while maintaining CD25 expression. Thus, it is possible to use CD38 to further improve the identification strategy of GC T_FR_ cells reported in this study as the vast majority of the T_FR_ cells that our method targets do not contain GC-resident cells. Nevertheless, a strategy for specifically capturing the most mature GC–resident CD25^−^ T_FR_ cells has yet to be established.

Overall, our results show that the close resemblance in surface receptors between T_FH_ and T_FR_ cells does not necessarily imply a parallel pattern of maturation for both cell types. Notably, the acquisition of CXCR5, ICOS, and PD-1 exhibits divergent trends along their maturation processes. The distinct usage of ICOS and PD-1 may provide an opportunity for selectively targeting T_FR_ cells at different stages of maturation.

## MATERIALS AND METHODS

### Experimental design

We hypothesized that the maturation of T_FH_ and T_FR_ cells follow distinct pathways. To investigate this, we studied these cell populations in human tonsils with the aim of elucidating their maturation processes through proteins previously known to be expressed by T_FH_ and T_FR_ cells. We used flow cytometry combined with a bioinformatics approach, specifically PCA, to analyze the dynamics of the proteins included in our staining panel simultaneously. Our initial observations indicated that the maturation of T_FH_ and T_FR_ cells, as assessed by the expression of PD-1 and ICOS, occurs in different patterns. To further validate these findings, we performed confocal imaging to confirm the spatial localization of distinct T_FR_ cell subsets: ICOS^−^PD-1^−^, ICOS^+^PD-1^−^, and ICOS^+^PD-1^+^. Because of the challenge of isolating T_FR_ cells from human tissues—largely owing to the lack of specific extracellular markers—and in light of our findings on the distinct maturation trajectories of T_FH_ and T_FR_ cells (based on PD-1 and ICOS expression), we developed an alternative sorting strategy. This method enables the isolation of a purer and more abundant population of T_FR_ cells from human lymphoid tissues.

### Human samples

Tonsils were collected from children submitted to tonsillectomy due to tonsil hypertrophy (*n* = 50, 3.8 ± 1.9 years old, 24 females and 26 males). Children with additional clinical conditions, or under any drug treatment, were excluded. The studies were approved by the Lisbon Academic Medical Center Ethics Committee (reference no. 548/14). Informed consent was obtained from all adult volunteers, parents, or legal guardians.

### Cell isolation

Lymphocytes from tonsils were isolated using Ficoll gradient medium (Histopaque-1077; Sigma-Aldrich) after mechanical disruption. Peripheral blood mononuclear cells (PBMCs) were isolated from buffy coats by Ficoll gradient medium using SepMate tubes (StemCell Technologies). Samples for cell sorting were previously treated with the MojoSort Human CD4 T Cell Isolation Kit (BioLegend) to separate CD4 T cell fractions: The CD4+ fraction was used to isolate CD4+ T cell subsets, whereas the CD4^−^ fraction was used to isolate naïve B cells (see fig. S8 for sorting strategy).

### Cell sorting and flow cytometry analysis

PBMCs and lymphocytes from tonsils were stained with the following antibodies for cell sorting and/or flow cytometry analysis: anti-CD4 (OKT4, BioLegend), anti-CD45RO (UCHL1, BioLegend), anti-CD185/CXCR5 (J252D4, BioLegend), anti-CD279/PD-1 (EH12.2H7, BioLegend), anti-CD278/ICOS (C398.4A, BioLegend), anti-CD27 (M-T271, BioLegend), anti-IgD (IA6-2, BioLegend), anti-CD38 (HB-7, BioLegend), anti-CD25 (BC96, Thermo Fisher Scientific), anti-CD127 (eBioRDR5, eBioscience), anti-CD19 (HIB19, eBioscience), anti-FOXP3 (PCH101, Thermo Fisher Scientific), and anti-BCL6 (K112-91, BD Biosciences). The Live/Dead Fixable Aqua Dead Stain Kit (Life Technologies) was used for exclusion of dead cells. Intracellular FOXP3 and BCL6 staining was performed using the FOXP3 Fix/Perm Kit (Thermo Fisher Scientific) according to the manufacturer’s instructions. Samples were acquired on a BD LSRFortessa cytometer (BD Biosciences) and further analyzed on the FlowJo software v.10.5.3 (TreeStar). Cell sorting was performed in a BD FACSAria Fusion instrument (BD Biosciences).

### Functional assays

For the in vitro functional assays, 30 × 10^3^ naïve B cells (CD19^+^IgD^+^CD27^−^) were cultured with 25 × 10^3^ T_FH_ cells (sorted as CD4^+^CD127^+^CD25^−^CXCR5^+^ in the blood and CD4^+^CD25^−^CXCR5^+^ICOS^+^ in the tonsil) and 25 × 10^3^ T_FR_ cells (sorted as CD4^+^CD127^−^CD25^+^CXCR5^+^ in blood and CD4^+^CD25^+^CXCR5^int^ICOS^int^ in tonsil). Cells were cultured with SEB (1 μg/ml) (Sigma-Aldrich). Cells were plated in U-bottom 96-well plates in RPMI 1640 (Life Technologies) supplemented with 10% heat-inactivated fetal bovine serum (Life Technologies), 1% Hepes (Sigma-Aldrich), 1% sodium pyruvate (Life Technologies), 1% penicillin-streptomycin (Life Technologies), and 0.05% gentamicin (Life Technologies) under 37°C and 5% CO_2_ incubator conditions. After 5 days, proliferation of T_FH_ cells was analyzed by using the CellTrace Violet (CTV) Proliferation Kit (Invitrogen) and differentiation of B cells was assessed through the up-regulation of CD38 by flow cytometry. For these functional assays, six biological replicates were used for the buffy coat experiments and four biological replicates for the tonsil experiments. Triplicates were performed for each biological replicate whenever cell numbers allowed. When cell numbers were limited, fewer technical replicates were conducted.

### Bioinformatics analysis

Fluorescence values were extracted for each marker (ICOS, PD-1, CD25, CXCR5, CD45RO, BCL6, and FOXP3) from flow cytometry data from tonsils by using the FlowJo software v.10.5.3 (TreeStar). For each tonsil, these values were extracted separately from different populations of T_FH_ and T_FR_ cells, as follows: PD-1^−^ICOS^−^, PD-1^−^ICOS^+^, PD-1^+^ICOS^−^, and PD-1^+^ICOS^+^ subsets. Further analysis was performed individually for each tonsil using the software RStudio (see fig. S9 for PCA in different tonsils). Principal components were calculated using the prcomp() function. Correlation matrices were calculated using the cor() function. Visualization of PCA and correlations was done by using the ggplot2 package. The analysis of T_FH_ cells combined PD-1^−^ICOS^−^ and PD-1^+^ICOS^+^ subsets, whereas for T_FR_ cells, the analysis included PD-1^−^ICOS^−^, PD-1^−^ICOS^+^, and PD-1^+^ICOS^+^ subsets (refer to fig. S10 for the combined analysis of T_FH_ and T_FR_ cells, irrespective of PD-1/ICOS subsets). This analysis was performed using R v.4.1.1.

### Immunofluorescence

Three-micrometer sections of formalin-fixed paraffin-embedded human tonsil were deparaffinized and submitted to antigen retrieval by heat (PT link pretreatment module for tissue specimens, Thermo Fisher Scientific at pH 9, Leica Biosystems). Background reduction was applied by incubation with 3% H_2_O_2_ (Sigma-Aldrich) in methanol. Total protein block was also applied (Dako). Then, sections were stained with anti-human FOXP3 (PCH101, Thermo Fisher Scientific), anti-human ICOS (D1K2T, Cell Signaling), and anti-human PD-1 (NAT105, BioLegend) primary antibodies. Anti-rat AF488 (Thermo Fisher Scientific), anti-rabbit AF546 (Thermo Fisher Scientific), and anti-mouse AF647 (Thermo Fisher Scientific) were used as secondary antibodies, respectively. Cell nuclei were visualized with 4′,6-diamidino-2-phenylindole (DAPI). Images were acquired on a Zeiss LSM 980 point-scanning confocal microscope with Airyscan 2, using a Plan-Apochromat 20x/0.8 objective (pixel size: 124 nm). Tile imaging and *z*-stack acquisition were used to cover the entire volume of interest. The tiled images were stitched together to reconstruct the full sample area, and subsequently, a maximum intensity projection was applied to generate a two-dimensional representation of the three-dimensional data. Cell counting was performed using software developed in-house, written in MATLAB (MathWorks, Natick, MA). Briefly, single cells were segmented and labeled with a pretrained Cellpose deep learning model (*32*) using the DAPI channel, and subsequent combinatorial filters for cell counting were defined on the basis of staining (e.g., positive staining in FOXP3 and ICOS channels but not in the PD-1 channel or positive staining in FOXP3, ICOS, and PD-1 channels), where a staining was considered positive if a minimum number of pixels were above a given threshold. Individual report images with segmentation and filtering results were also generated by the software for cell counting verification. GC boundaries were defined based on cell density as visualized by DAPI nuclear staining (fig. S11).

### Statistical analysis

The Shapiro-Wilk normality test was applied to assess the normality of the data distribution. For comparisons between two groups, the paired *t* test was used if data passed the normality test. If the data did not meet the normality assumption, the Wilcoxon matched-pairs signed-rank test was applied. For pairwise multiple comparisons, either the Friedman test with Dunn’s multiple comparisons test or the two-way analysis of variance (ANOVA) with Bonferroni’s multiple comparison test were applied. For the functional assays, the Wilcoxon matched-pairs signed-rank test was used to assess statistical significance within the blood and tonsil, whereas the unpaired Mann-Whitney *U* test was applied to assess differences between compartments. *P* values less than 0.05 were considered significant. Graphs were prepared using GraphPad Prism v.8.4.3 software, and statistical analysis was performed using GraphPad Prism v.8.4.3 and R studio v.4.3.0.
