# Supplementary material for: PD-1 and ICOS are coexpressed in T follicular helper cells but define three stages of maturation of T follicular regulatory cells
Source: Sci Adv. 2025 Jul 11;11(28):eadt8901. doi: 10.1126/sciadv.adt8901 (PMC12248373; doi:10.1126/sciadv.adt8901)
Supplement: Supplementary file 1 — Figs. S1 to S11 Table S1 Legend for table S2 [file sciadv.adt8901_sm.pdf]

Science Advances
AAAS

Supplementary Materials for

PD-1 and ICOS are coexpressed in T follicular helper cells but define three stages of maturation of T follicular regulatory cells

Filipa Ribeiro *et al.*

Corresponding author: Luis Graca, lgraca@medicina.ulisboa.pt

Sci. Adv. **11**, eadt8901 (2025)
DOI: 10.1126/sciadv.adt8901

The PDF file includes:

Figs. S1 to S11
Table S1
Legend for table S2

Other Supplementary Material for this manuscript includes the following:

Table S2

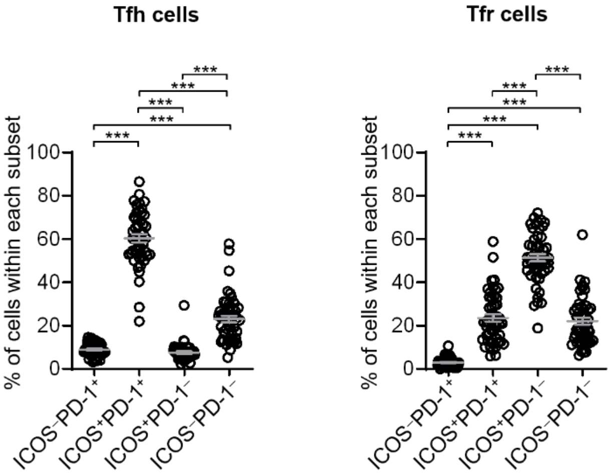

Fig. S1. ICOS and PD-1 subsets within FOXP3^{-}CXCR5^{+}CD25^{-} Tfh and FOXP3^{+}CXCR5^{+} Tfr cells. Distribution of ICOS^{-}PD-1^{+}, ICOS^{+}PD-1^{+}, ICOS^{+}PD-1^{-}, and ICOS^{-}PD-1^{-} subsets within FOXP3^{-}CXCR5^{+}CD25^{-} Tfh and FOXP3^{+}CXCR5^{+} Tfr cells. Tfh cells were divided into two major subpopulations (ICOS^{+}PD-1^{+} and ICOS^{-}PD-1^{-}), while Tfr cells were categorized into three major subpopulations (ICOS^{+}PD-1^{+}, ICOS^{+}PD-1^{-}, and ICOS^{-}PD-1^{-}) for further analysis (n=50). Error bars represent mean ± SEM. ***p<0.001 using Friedman test with Dunn's multiple comparisons test.

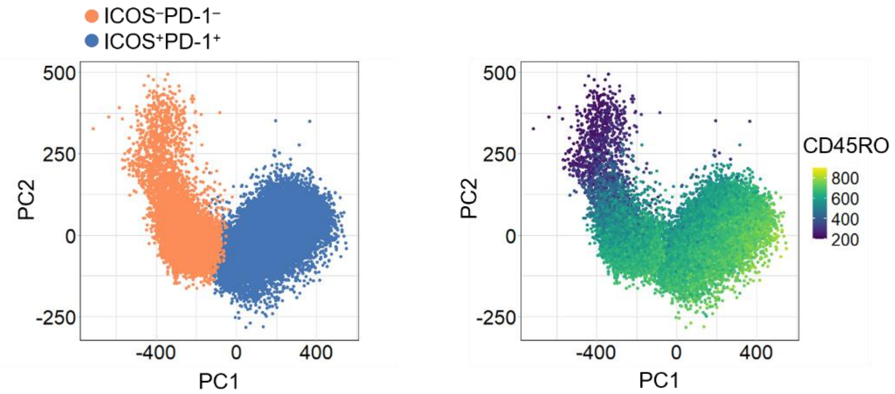

Fig. S2. PC2 in Tfh cell analysis segregates CD45RO^{+} and CD45RO^{-} populations. PCA of the fluorescence values exported from the flow cytometry data in Tfh cells show that PC2 is explained by the differential expression of CD45RO in ICOS^{-}PD-1^{-} and ICOS^{+}PD-1^{+} cell subsets.

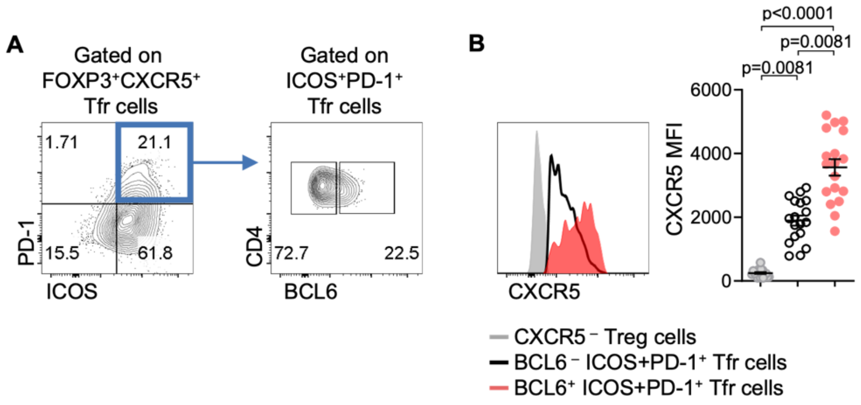

Fig. S3. CXCR5 expression within BCL6⁻ICOS⁺PD-1⁺ and BCL6⁺ICOS⁺PD-1⁺ Tfr cell compartments. (A) Representation of how cells were selected as BCL6⁺ and BCL6⁻ cells. **(B)** Histograms of the distribution of CXCR5 among BCL6⁻ICOS⁺PD-1⁺ Tfr cells (black line) and BCL6⁺ICOS⁺PD-1⁺ Tfr cells (red). CXCR5⁻ Treg cells (selected as CD4⁺FOXP3⁺CXCR5⁻; gray) were used as a negative control for CXCR5 expression. Representative plots (left) and pooled data (right) (n=18). Error bars represent mean ± SEM. Friedman test with Dunn's multiple comparisons test was applied.

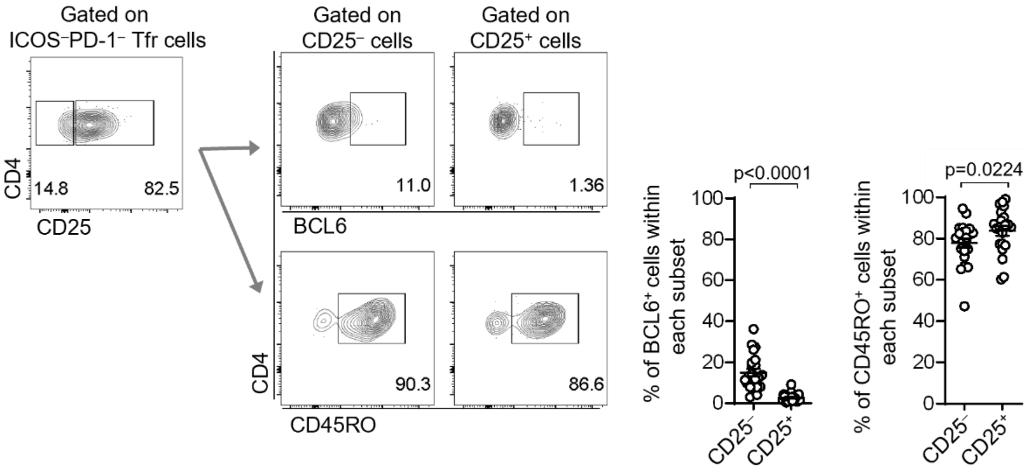

Fig. S4. Expression of BCL6 and CD45RO within ICOS⁻PD-1⁻ Tfr cells. Expression of BCL6 (top) and CD45RO (below) within ICOS⁻PD-1⁻ Tfr cells either expressing CD25 (right) or not (left). Representative flow cytometry plots (left) and pooled data (right) are shown (n=22). Error bars represent mean ± SEM. Wilcoxon matched-pairs signed rank test was applied.

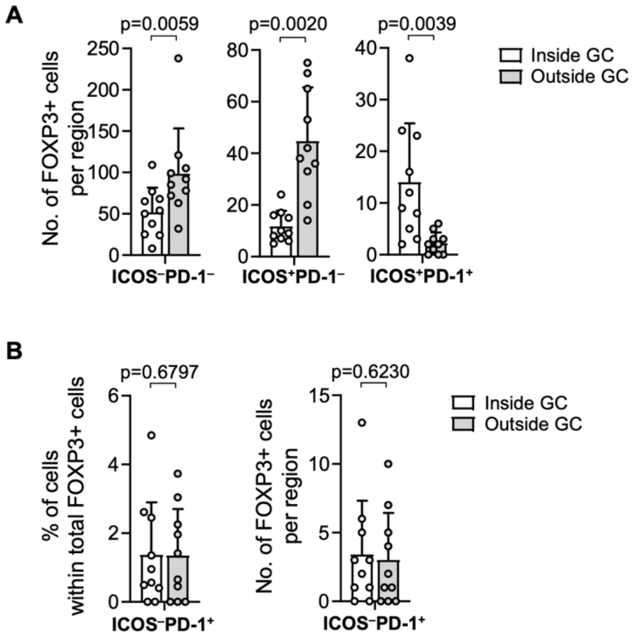

Fig. S5. Quantification of Tfr cell subsets within and outside the GC. (A) Number of ICOS$^-$PD-1$^-$, ICOS$^+$PD-1$^-$ and ICOS$^+$PD-1$^+$ Tfr cells within (white bars) and outside (gray bars) the GC. **(B)** Frequency within total FOXP3$^+$ cells (left) and number (right) of ICOS$^-$PD-1$^+$ cells within (white bars) and outside (gray bars) the GC. Data are pooled from 2-3 images per tonsil of four healthy children (n=10). Error bars represent mean ± SEM. Wilcoxon matched-pairs signed rank test was applied.

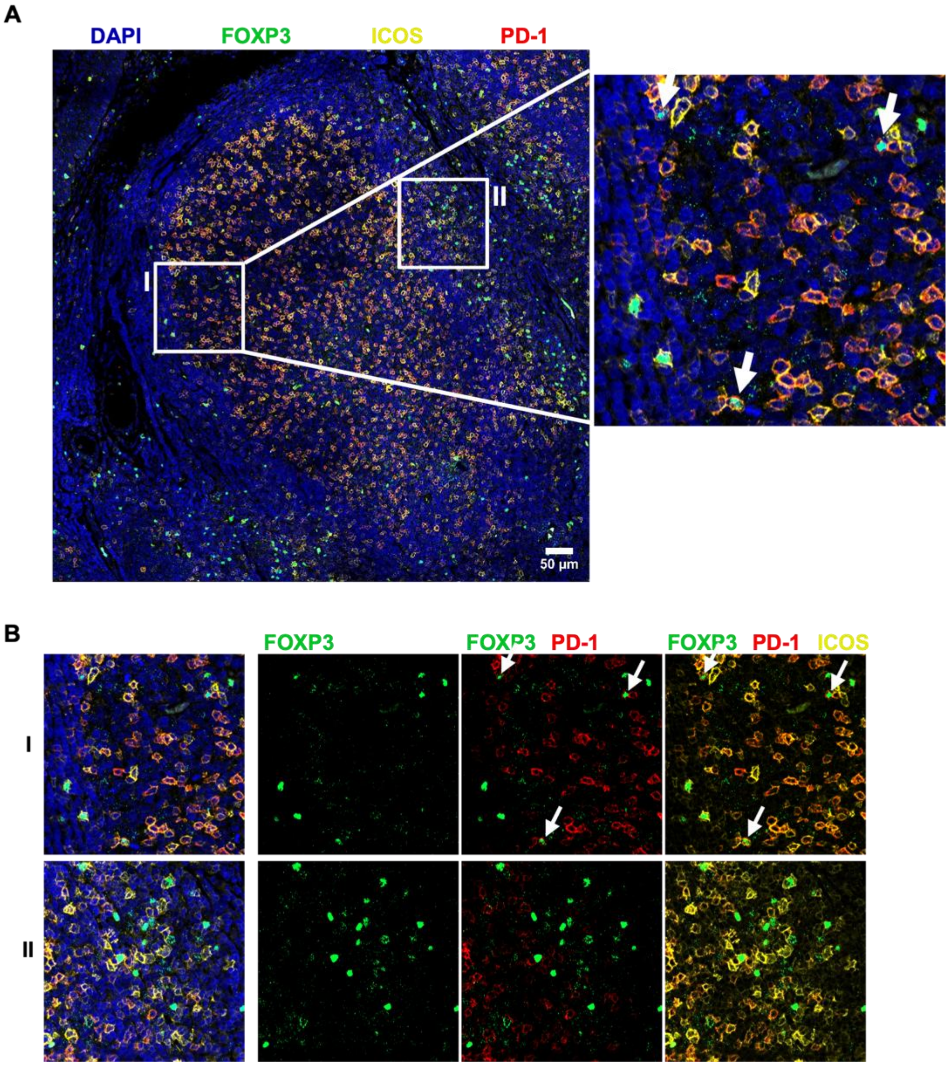

Fig. S6. ICOS$^+$PD-1$^+$ Tfr cells localize specifically within the GC. (A) Another representative image of immunofluorescence microscopy of human tonsils stained for DAPI (blue), FOXP3 (green), ICOS (yellow) and PD-1 (red). Individual image panels were stitched together to reconstruct the full sample area using tile imaging and z-stack acquisition. Outlined area I is represented as an enlarged image on the right, in which ICOS$^+$PD-1$^+$FOXP3$^+$ cells are identified (white arrows). **(B)** Outlined areas I and II indicated in (A). Data are representative of tonsil sections from four healthy children.

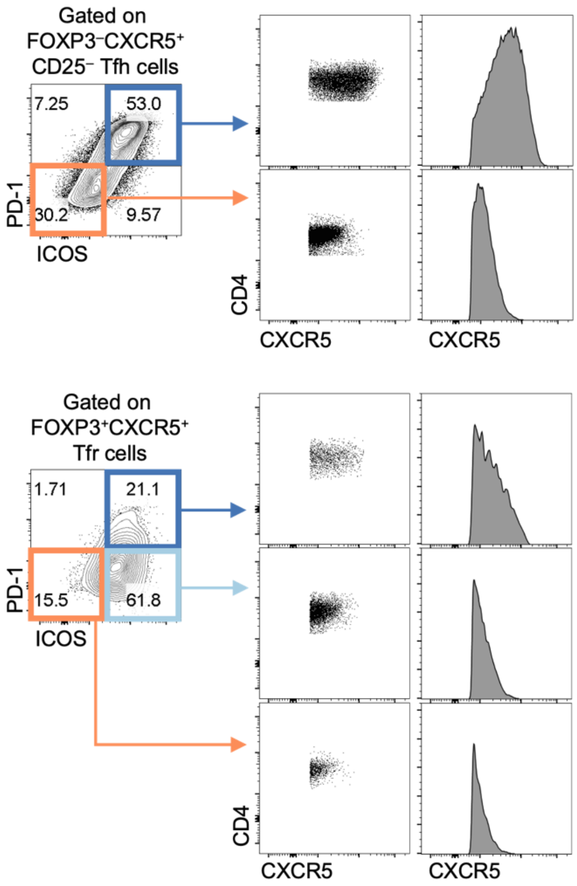

Fig. S7. CXCR5 expression in distinct subpopulations of Tfh and Tfr cells. Expression levels of CXCR5 within ICOS$^+$PD-1$^+$ (blue) and ICOS$^-$PD-1$^-$ (orange) subpopulations of Tfh (top) and Tfr (bottom) cells from human tonsil. For Tfr cells, the expression of CXCR5 within ICOS$^+$PD-1$^-$ subset (light blue) is also shown.

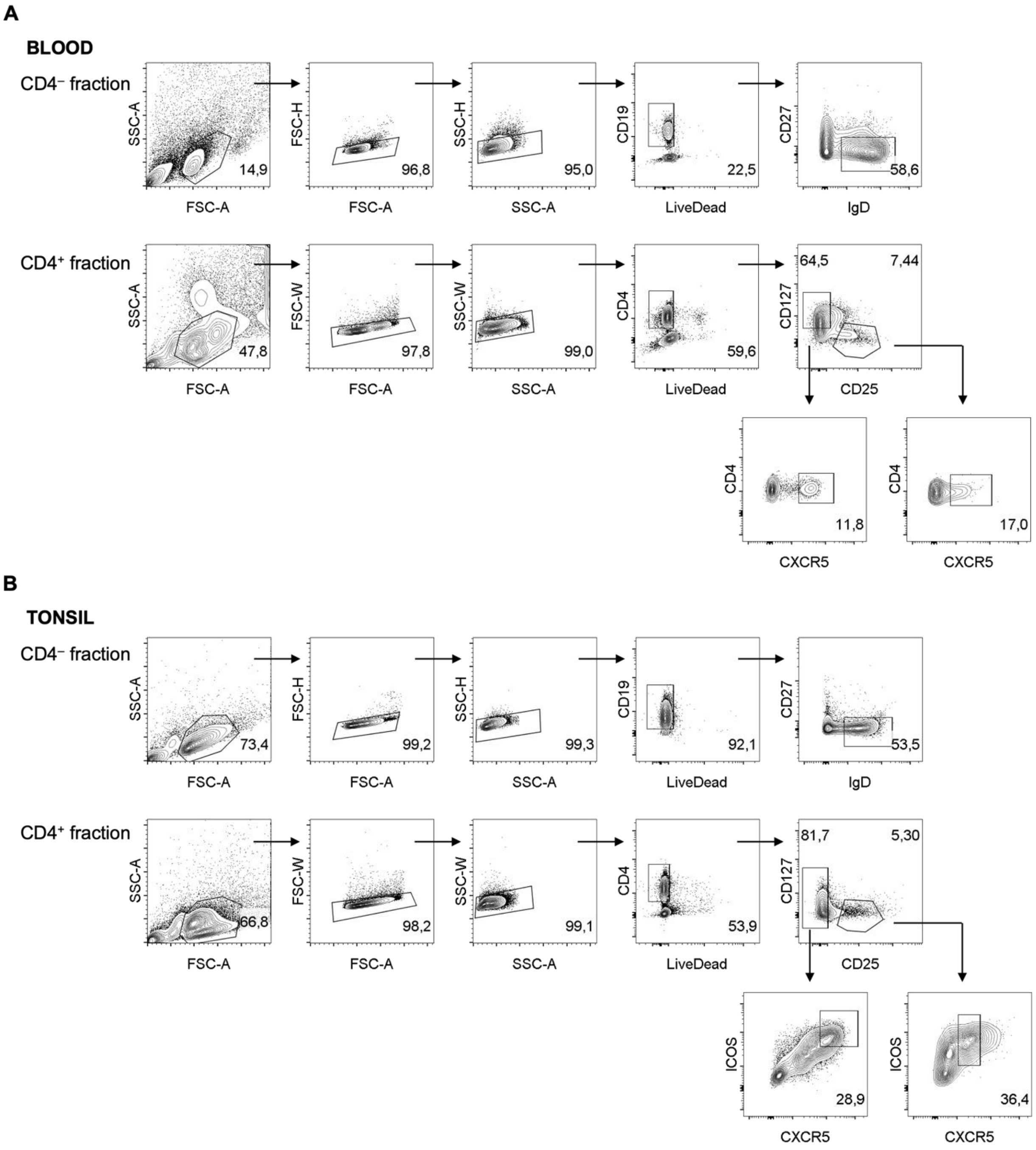

Fig. S8. Sorting strategy for isolation of human naïve B cells, Tfh cells and Tfr cells from blood and tonsil. (A) Sorting strategy for isolation of $CD19^{+}IgD^{+}CD27^{-}$ naïve B cells (top), $CD4^{+}CD127^{+}CD25^{-}CXCR5^{+}$ Tfh cells and $CD4^{+}CD127^{-}CD25^{+}CXCR5^{+}$ Tfr cells (below) from peripheral blood (buffy-coat). **(B)** Sorting strategy for isolation of $CD19^{+}IgD^{+}CD27^{-}$ naïve B cells (top), $CD4^{+}CD25^{-}CXCR5^{+}ICOS^{+}$ Tfh cells and $CD4^{+}CD25^{+}CXCR5^{int}ICOS^{int}$ Tfr cells (below) from tonsils.

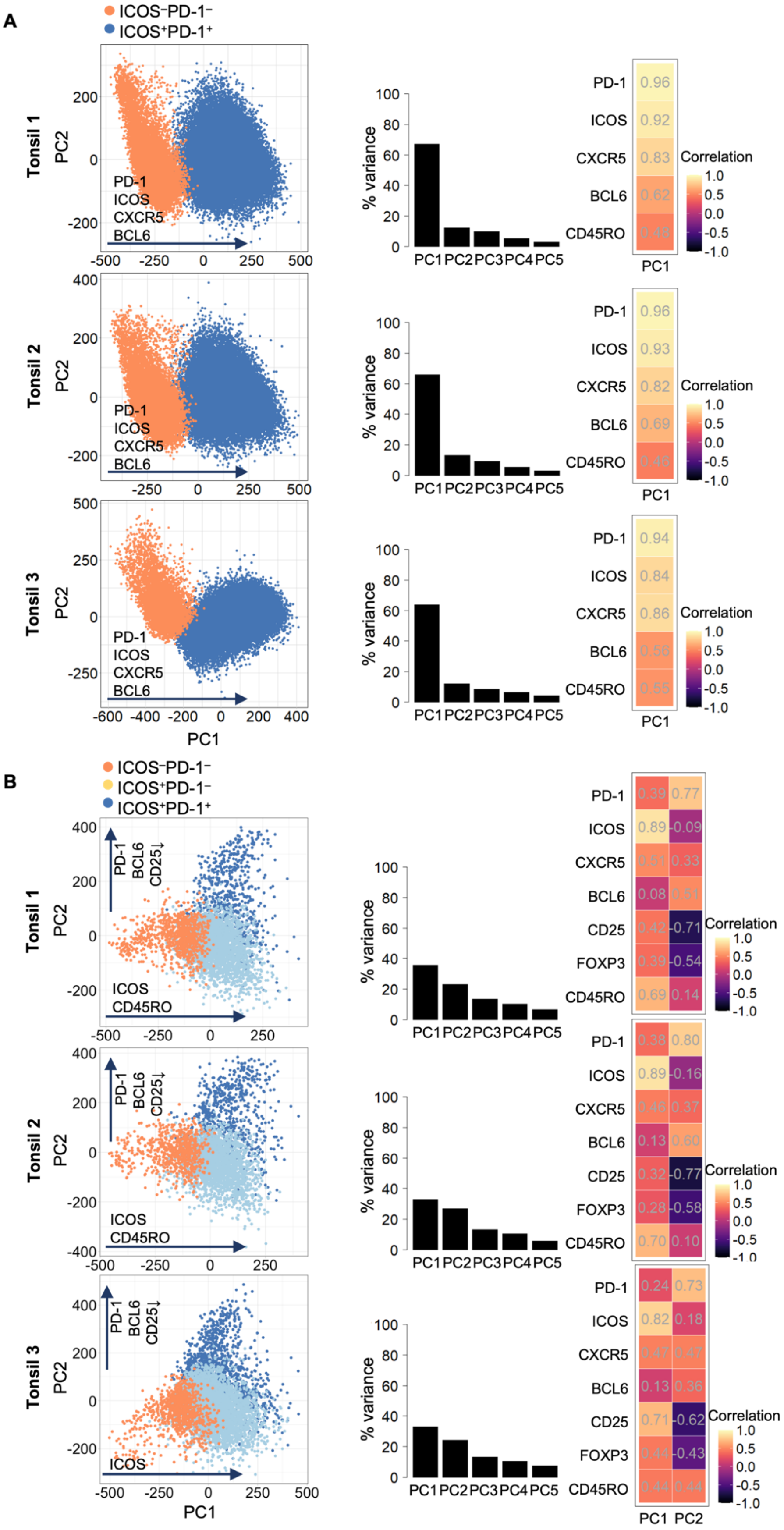

A
ICOS⁻PD-1⁻
ICOS⁺PD-1⁺
Tonsil 1
Tonsil 2
Tonsil 3
PC2
PC1
PD-1
ICOS
CXCR5
BCL6
% variance
PC1 PC2 PC3 PC4 PC5
PD-1 0.96
ICOS 0.92
CXCR5 0.83
BCL6 0.62
CD45RO 0.48
Correlation
PD-1 0.96
ICOS 0.93
CXCR5 0.82
BCL6 0.69
CD45RO 0.46
PD-1 0.94
ICOS 0.84
CXCR5 0.86
BCL6 0.56
CD45RO 0.55
B
ICOS⁻PD-1⁻
ICOS⁺PD-1⁻
ICOS⁺PD-1⁺
PD-1
BCL6
CD25↓
ICOS
CD45RO
PD-1 0.39 0.77
ICOS 0.89 -0.09
CXCR5 0.51 0.33
BCL6 0.08 0.51
CD25 0.42 -0.71
FOXP3 0.39 -0.54
CD45RO 0.69 0.14
PD-1 0.38 0.80
ICOS 0.89 -0.16
CXCR5 0.46 0.37
BCL6 0.13 0.60
CD25 0.32 -0.77
FOXP3 0.28 -0.58
CD45RO 0.70 0.10
PD-1 0.24 0.73
ICOS 0.82 0.18
CXCR5 0.47 0.47
BCL6 0.13 0.36
CD25 0.71 -0.62
FOXP3 0.44 -0.43
CD45RO 0.44 0.44

Fig. S9. PCA of Tfh and Tfr cells from three different tonsils. (A) PCA of the FOXP3^{-}CXCR5^{+}CD25^{-} Tfh cell subsets (ICOS^{-}PD-1^{-} in orange; ICOS^{+}PD-1^{+} in blue) based on the fluorescence values of each flow cytometry marker (left); percentage of variance of the data explained by the first five principal components (middle); and heatmap of the correlation values for each of the markers explained by PC1 (right). **(B)** PCA of the FOXP3^{+}CXCR5^{+} Tfr cell subsets (ICOS^{-}PD-1^{-} in orange; ICOS^{+}PD-1^{-} in light blue; ICOS^{+}PD-1^{+} in blue) based on the fluorescence values of each flow cytometry marker (left); percentage of variance of the data explained by the first five principal components (middle); and heatmap of the correlation values for each marker explained by PC1 and PC2 (right). Analysis of different tonsils is shown (n=3).

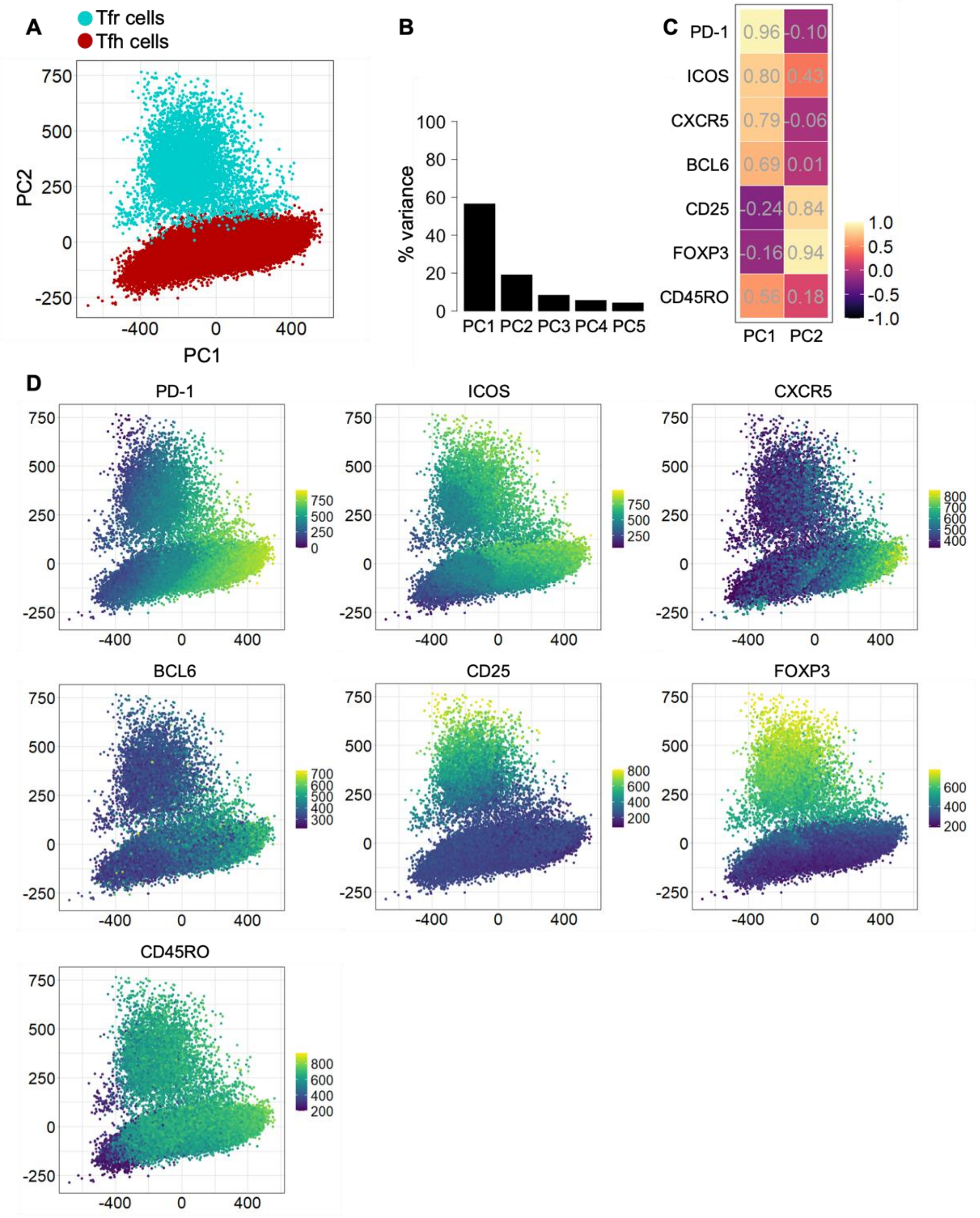

Fig. S10. Combined analysis of Tfh and Tfr cells, irrespective of PD-1/ICOS subsets. (A) PCA of FOXP3$^-$CXCR5$^+$CD25$^-$ Tfh cells (red) and FOXP3$^+$CXCR5$^+$ Tfr (blue) cells analyzed together, considering all cell populations, regardless of the expression of ICOS and/or PD-1. **(B)** Percentage of variance of the data explained by the first five principal components. **(C)** Heatmap of the correlation values for each marker explained by PC1 and PC2. **(D)** Expression of each marker in the PCA plot. PC1 is the component that contributes the most to the variance (56.52%), by explaining a strong maturation axis of Tfh and Tfr cells given the upregulation of PD-1, ICOS, CXCR5, BCL6 and CD45RO. In addition to segregating the regulatory and non-regulatory cell populations, PC2 also accounts for the variance in ICOS expression.

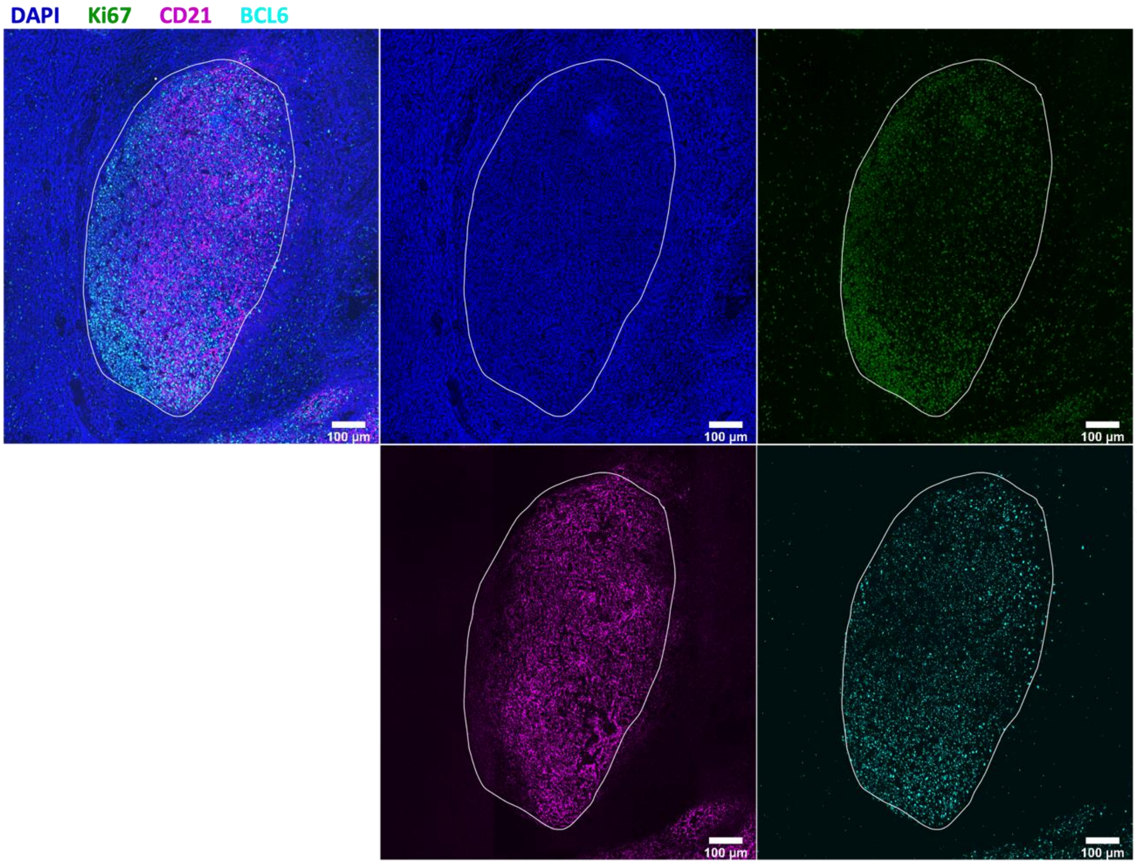

Fig. S11. Validation of DAPI-based GC delineation in human tonsil. Immunofluorescence images showing a merged image of DAPI, Ki67, CD21, and BCL6), and individual channels for each GC marker. White lines indicate the GC boundaries determined by DAPI-based cell density. The delineation derived from DAPI corresponds closely with the distribution patterns of GC-specific markers, supporting the use of nuclear density for GC identification when other markers cannot be used. Scale bar: 100 µm.

Table S1. Commercial reagents.
This table contains the information of the commercial reagents used for the experiments described in the manuscript.

Reagent	Supplier	Catalogue	RRID
Histopaque®-1077	Sigma-Aldrich	#10771	-
SepMate™ tubes	StemCell Technologies	#85460	-
MojoSort™ Human CD4 Nanobeads	BioLegend	#480014	-
Brilliant Violet 711™ anti-human CD4 Antibody	BioLegend	#317440	AB_2562912
Brilliant Violet 605™ anti-human CD279 (PD-1) Antibody	BioLegend	#329924	AB_2563212
PerCP/Cyanine5.5 anti-human/mouse/rat CD278 (ICOS) Antibody	BioLegend	#313518	AB_10641280
APC/Cyanine7 anti-human CD27 Antibody	BioLegend	#302816	AB_571977
Brilliant Violet 785™ anti-human IgD Antibody	BioLegend	#348242	AB_2629809
PE anti-human CD38 Antibody	BioLegend	#356604	AB_2561900
CD25 Monoclonal Antibody (BC96), PE-Cyanine7, eBioscience™	Thermo Fisher Scientific	#25-0259-42	AB_1257140
CD127 Monoclonal Antibody (eBioRDR5), APC-eFluor™ 780, eBioscience™	Thermo Fisher Scientific	#47-1278-42	AB_1548674
PE/Cyanine7 anti-human CD19 Antibody	BioLegend	#302216	AB_314246
FOXP3 Monoclonal Antibody (PCH101), eFluor™ 450, eBioscience™	Thermo Fisher Scientific	#48-4776-42	AB_1834364
BD Pharmingen™ Alexa Fluor® 488 Mouse anti-Bcl-6	DB Biosciences	#561524	AB_10716202
LIVE/DEAD™ Fixable Aqua Dead Cell Stain Kit	Life Technologies	#L34957	-
FOXP3 Fix/Perm Kit	Thermo Fisher Scientific	#00-5521-00	-
Staphylococcal enterotoxin B from Staphylococcus aureus	Sigma-Aldrich	#S4881-1MG	-
RPMI 1640 Medium, GlutaMAX™ Supplement	Life Technologies	#61870010	
Fetal Bovine Serum, Value, heat inactivated	Life Technologies	#A5256801	-
HEPES solution	Sigma-Aldrich	#H0887	-
Sodium pyruvate solution	Life Technologies	#11360039	-

Penicillin-Streptomycin (10,000 U/mL)	Thermo Fisher Scientific	#15140122	-
Gentamicin (50 mg/mL)	Thermo Fisher Scientific	#15750037	-
CellTrace™ Violet Cell Proliferation Kit	Invitrogen	#C34557	-
FOXP3 Monoclonal Antibody (PCH101), eBioscience™	Thermo Fisher Scientific	#14-4776-82	AB_467554
ICOS (D1K2T™) Rabbit mAb	Cell Signaling	#89601S	
Purified anti-human CD279 (PD-1) Antibody	BioLegend	#367402	AB_2565782
Donkey anti-Rat IgG (H+L) Highly Cross-Adsorbed Secondary Antibody, Alexa Fluor™ 488	Thermo Fisher Scientific	#A-21208	AB_2535794
Goat anti-Rabbit IgG (H+L) Cross-Adsorbed Secondary Antibody, Alexa Fluor™ 546	Thermo Fisher Scientific	#A-11010	AB_2534077
Goat anti-Mouse IgG (H+L) Cross-Adsorbed Secondary Antibody, Alexa Fluor™ 647	Thermo Fisher Scientific	#A-21235	AB_2535804
DAPI	Thermo Fisher Scientific	#00-4959-52	-

Table S2. Raw data for cytometry and miscroscopy analyses (separate file).
Excel file containing raw data from cytometry and microscopy experiments.
